# The Functions and Mechanisms of Basic Fibroblast Growth Factor in Tendon Repair

**DOI:** 10.3389/fphys.2022.852795

**Published:** 2022-06-13

**Authors:** Jingwei Lu, Li Jiang, Yixuan Chen, Kexin Lyu, Bin Zhu, Yujie Li, Xueli Liu, Xinyue Liu, Longhai Long, Xiaoqiang Wang, Houping Xu, Dingxuan Wang, Sen Li

**Affiliations:** ^1^ School of Physical Education, Southwest Medical University, Luzhou, China; ^2^ The Affiliated Traditional Chinese Medicine Hospital, Southwest Medical University, Luzhou, China

**Keywords:** basic fiboblast growth factor, tendon injury, tendon repair, tendon healing, mechanism

## Abstract

Tendon injury is a disorder of the musculoskeletal system caused by overuse or trauma, which is characterized by pain and limitations in joint function. Since tendon healing is slowly and various treatments are generally ineffective, it remains a clinically challenging problem. Recent evidences suggest that basic fibroblast growth factor (bFGF) not only plays an important role in tendon healing, but also shows a positive effect in laboratory experimentations. The purpose of this review is to summarize the effects of bFGF in the tendon healing. Firstly, during the inflammatory phase, bFGF stimulates the proliferation and differentiation of vascular endothelial cells to foster neovascularization. Furthermore, bFGF enhances the production of pro-inflammatory factors during the early phase of tendon healing, thereby accelerating the inflammatory response. Secondly, the cell proliferation phase is accompanied by the synthesis of a large number of extracellular matrix components. bFGF speeds up tendon healing by stimulating fibroblasts to secrete type III collagen. Lastly, the remodeling phase is characterized by the transition from type III collagen to type I collagen, which can be promoted by bFGF. However, excessive injection of bFGF can cause tendon adhesions as well as scar tissue formation. In future studies, we need to explore further applications of bFGF in the tendon healing process.

## Introduction

Tendon is a special connective tissue linking muscle with bone, which consists of a vast array of collagen fiber bundles (Mienaltowski and Birk, 2014). Overuse and trauma are the main causes of tendon injuries, which account for approximately 30% of musculoskeletal disorders (Kaux et al., 2011; Nourissat et al., 2015). However, due to the special characteristics of tendons with poor vascularity and low metabolic rate, the healing process of injuries is very slow and the quality of repair is poor (Sakabe and Sakai, 2011; Nourissat et al., 2015). Therefore, it is of particular importance that treatment of tendon injury focuses on speeding up tendon healing and improving the quality of healing.

At present, the treatment of tendon injuries is categorized into surgical and non-surgical treatments. In many cases, the preferred treatment modality is generally non-surgical, with surgery being considered the alternative of last option (Docheva et al., 2015). Non-surgical treatments include drug therapy, injection therapy, exercise therapy, growth factor therapy, stem cells therapy, physical therapy, etc. (Yang and Chen, 2020). There are two situations in which tendon injuries require surgical treatment, either when the tendon is ruptured or when conservative treatment is ineffective. However, there are still disadvantages to surgical treatment, which can easily induce complications and inflammation, thereby in turn affecting the effectiveness of the tendon repair (Jallageas et al., 2013; Snedeker and Foolen, 2017b).

As we all know, bFGF is a mitogenic factor isolated and purified from bovine brain, which has an important role in wound healing and cell proliferation (including vascular endothelial cells, fibroblasts, nerve cells, and tendon cells), and osteogenesis (Hsu and Chang, 2004). Recently investigators have examined the effects of bFGF on the process of tendon repair. The investigators found that bFGF injection significantly accelerated tendon healing and also discovered that bFGF was active in all three phases of tendon repair, besides improving the ultimate strength of repaired tendon and reducing the adhesions after healing (Moshiri and Oryan, 2011; Oryan and Moshiri, 2014; Najafbeygi et al., 2017; Zhou et al., 2021). Moreover, injection of exogenous bFGF not only induces endogenous bFGF to synergistically interact with it to contribute to tendon healing, but also provokes other GFs to rise (Hamada et al., 2006; Wang et al., 2014). In addition, compared with other treatment methods, bFGF therapy has the merit of low recurrence rate and improved biomechanical function of the repaired tendon (Oryan and Moshiri, 2011; Madry et al., 2013).

Multiple studies have illustrated the role of bFGF in tendon repair, however, the detailed mechanisms have not been fully elucidated. Therefore, the purpose of this review is to summarize the role and mechanisms of bFGF in the three stages of tendon healing, to further explore the ways in which bFGF can be used in the future treatment of tendon injuries, and to provide a theoretical foundation for subsequent researchers.

**FIGURE 1:**
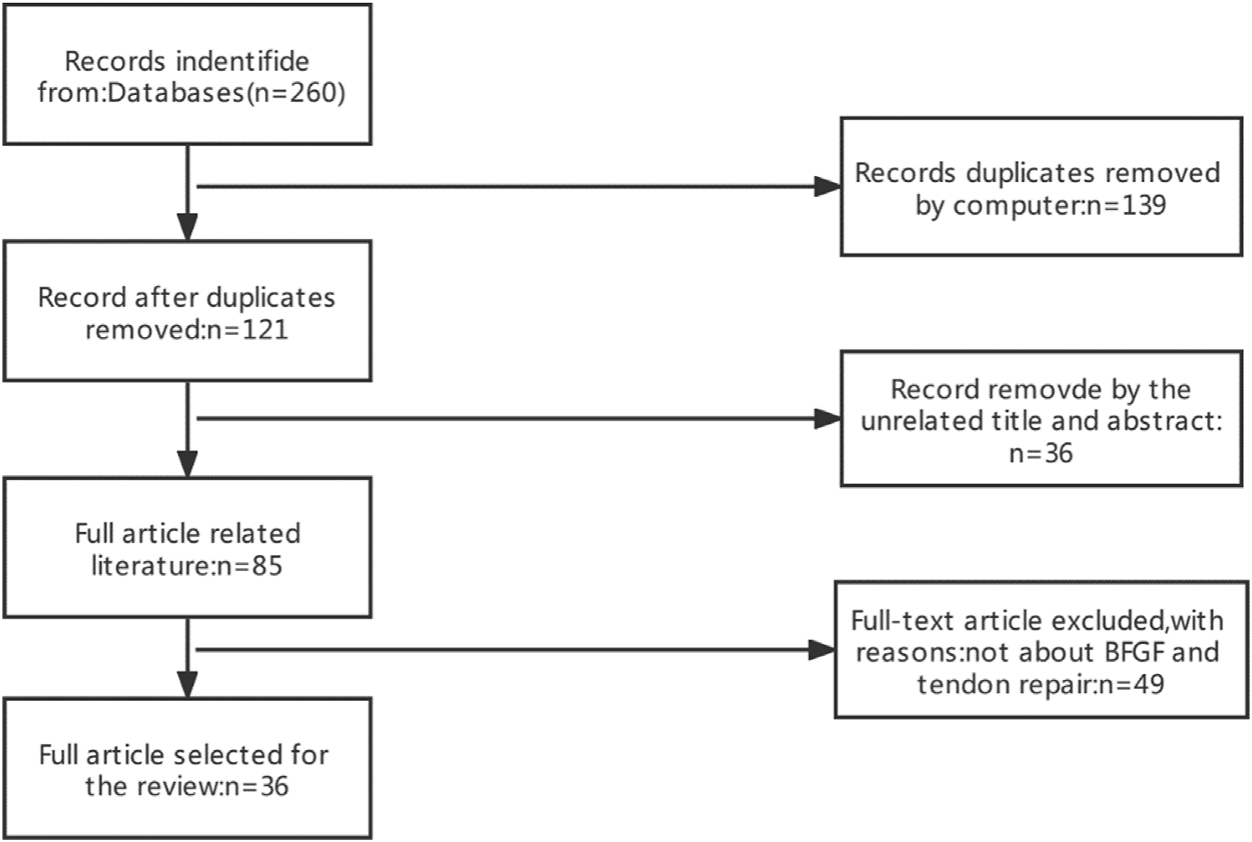
Article Retrieval Flow Chart with inclusion and exclusion process.

Search strategy: 1) Search site: Articles are from PubMed, a database of papers on biomedical science. 2) Database: MEDLINE. 3) Keywords: basic fibroblast growth factor, BFGF, tendon injury, tendinopathy, tendon repair 4) Boolean algorithm: (“Basic fibroblast growth factor” OR “bFGF” OR “FGF2”) AND (“Tendinopathy” OR “Tendon injuries” OR “Tendon repair”). 5) Retrieval timeframe: We searched the selected journals published from 1991 to 2021. 6) Inclusion and exclusion criteria: Articles were included if the topic is related to basic fibroblast growth factor and tendon repair, and the article type was a review or an experimental paper. The search process was performed as presented in Figure 1.

## Basic Fibroblast Growth Factor in Tendon Healing: Experimental Studies

Many researches have shown that bFGF has an important role in tendon healing process (Kataoka et al., 2021; Yan et al., 2021). Related experiments regarding the effect of bFGF (or viruses and plasmids carrying the bFGF gene) on tendon healing are summarized in Table 1.

**TABLE 1:** Summary of results and characteristics of the studies which investigated the effects of bFGF in tendon healing.

Study	Animal type	Models establish	Dosage	Time post operation	Outcome	Conclusion
Chan et al. (2000)	Sprague Dawley rats	Patellar tenotomy	bFGF: 0, 10, 100, 1000 ng/ml	1, 2 weeks	Type III collagen↑ ultimate stress n.a	The injection of bFGF promotes cell proliferation and the synthesis of type III collagen in the early stage of tendon healing, but without improving ultimate strength
					Number of cells↑	
Oryan and Moshiri, (2011)	New Zealand rabbits	SDFT tenotomy and repair	Human recombinant bFGF: 1200 ng/kg	3, 7, 10 days	collagen fibrils diameter↑Biomechanical properties↑Clinical observations↑	Administration of human recombinant bFGF can significantly enhance the structural and biomechanical of tendon, and prevent tendon adhesion
Thomopoulos et al. (2010)	Mongrel dogs	flexor tendon transection and repair	bFGF:either 500 ng (1.25 mg/ml)	21 days	Blood vessel	Administration of bFGF leads to increased tendon cellularity and matrix synthesis. But did not improve the mechanical properties of the tendon
			or 1000 ng (2.5 mg/ml)		Density↑cellularity↑ratio of type-I collagen to type-III collagen↓tensile mechanical properties n.a	
Tang et al. (2008)	white leghorn chickens	FDP tenotomy and repair	bFGF: 2 × 10^9^viral particles	2, 4, 8, 12 weeks	Ultimate Strength↑strength of tendons↑gliding function↑grading of adhesions↓	AAV2-bFGF improved healing strength without aggravating
						adhesion formation after tendon injury
Hamada et al. (2006)	Japanese white rabbits	left FDFT tenotomy and repair	bFGF: 400 μg/ml	1, 3, 6 weeks	mechanical strength↑collagen	This bFGF-coated nylon suture induced an increase of biomechanical strength and accelerated cellular proliferation
					and proliferation of tenocytes↑	
Wang et al. (2005)	*In vitro*	Tenocytes of rat intrasynovial tendons	AAV2-bFGF	10 days	Type I collagen↑Type IIIcollagen↑	Delivery of exogenous bFGF gene to tenocytes can increase significantly the levels of expression of the bFGF and type I and III collagen genes
					Cell proliferation↑	
Chang et al. (1998)	New Zealand white rabbit	DFTP transection and repair	NA	1, 3, 7, 14, 28, 56 days	Vascular numbers ↑	bFGF can promote angiogenesis in the early stage of tendon healing
Tokunaga et al. (2015a)	Adult male Sprague-Dawley rats	supraspinatus tendon transection and repair	gelatin hydrogel	2, 4, 6, 8, 12 weeks	MSCs↑biomechanical strength↑fibrovascular scarring↓Scx↑collagen fibers↑	Administration of FGF-2 stimulated the growth of fibroblastic progenitor cells, resulting in biomechanical and histological improvements of the repaired RC
			sheets containing recombinant human FGF-2: 5 µg			
Tang et al. (2014)	white Leghorn chick-ens	FDP tenotomy and repair	bFGF: 2 × 10^9^viral particles	2, 3, 4, 5, 6, 7, 8 weeks	TGFβ↑VEGF↑CTGF↑ PDGF n.a type I collagen↑	Introduction of the FGF-2 gene will lead to the expression of other growth factor genes, thus promoting tendon repair
Kobayashi et al. (1997)	adult mongrel ca-nines	ACL laceration and repair	recombinant human bFGF: 10 µg	1, 3, 6, 24 weeks	The number of newly formed vessels ↑granulation tissue↑	the application of a bFGF-impregnated
						pellet seems to enhance the healing potential of the par-tially lacerated ACL

↑, significant increase; ↓, significant decrease.

n.a, not affected, NA, not applicable; VEGF, vascular endothelial growth factor; CTGF, connective tissue growth factor; TGF-β, transforming growth factor-β; PDGF, platelet-derived growth factor; bFGF, Basic fibroblast growth factor; FDP, flexor digitorum profundus; AAV2, adeno-associated viral type-2; SH, sodium hyaluronate; MCL, mean color level; RC, rotator cuff; Scx, Scleraxis; MSCs, mesenchymal stem cells.

Kobayashi et al. (1997) studied the effect of bFGF application on ACL lacerations in adult mongrel canines and then found that the number of blood vessels and granulation tissue formation were significantly higher in wounds sutured with bFGF-impregnated granules than in controls. This was one of the reasons for promoting early healing of the ACL. Chang et al. (1998) established a model of rabbit flexor tendon wound healing with the aim of studying the expression of bFGFmRNA at the injury site. In this study, the neovascularization at the repair site was accompanied by the upregulation of bFGFmRNA.

In addition, the advantages of exogenous bFGF were also investigated in (Chan et al., 2000), and (Wang et al., 2005). Chan et al. (2000) created a rat patellar injury model and found that bFGF injection significantly enhanced cell proliferation and type III collagen expression in the injured area. The number of cell proliferation and the level of type III collagen expression showed a dose-dependent increase at 7 days after injury. However, bFGF was not found to have an effect on ultimate stress in this study. on the contrary, (Tang et al., 2008) injections of AAV2 bFGF into both ends of the severed flexor tendons from white leghorn chickens were found to promote tendon ultimate stress without increasing adhesion formation. Furthermore, Wang et al. (2005) transferred exogenous bFGF gene into proliferating tenocytes via adeno-associated viral 2 (AAV 2) vectors. As a result, they found that AAV2 bFGF significantly increased the expression of bFGF, type I collagen and type III collagen genes. Similarly, Tang et al. (2014) found that injection of AAV2 bFGF evoked a significant upregulation of other growth factors in chicken flexor tendons.

In contrast to others, (Hamada et al., 2006) monofilament nylon thread in bFGF solution and then used it to suture injured flexor tendons in Japanese white rabbits. The results indicated that bFGF-coated nylon suture induced an increase of biomechanical strength as well as cell proliferation in rabbit flexor tendons.

Thomopoulos et al. (2010) established a canine model of flexor tendon injury with second- or fifth-digit repair of the right forelimb, after surgery, with two different doses (500 and 1000 ng) of bFGF matrix protein compared with operative repair. Compared with tendons that received operative repair alone, vascular, cellular, and adhesion formation increased with higher doses of bFGF, and adhesions become more severe with higher doses. However, there was no significant improvement in tensile mechanical properties. Conversely, Oryan and Moshiri, (2011) studied the effect of bFGF on the rupture of finger flexor tendons in rabbits after surgical repair. It was found that the biomechanical properties of tendons injected with bFGF were significantly improved, along with increased cell numbers and collagen production compared to controls, without an additional peritendinous adhesion. Similarly, Tokunaga et al. (2015a) disposed gelatin hydrogels containing 5 mg of FGF-2 into the post-transection supraspinatus tendons of rats. Biomechanical analysis of the specimens at 6 and 12 weeks revealed a remarkable increase in ultimate load in the FGF2-treated group.

Finally, Najafbeygi et al. (2017) established a New Zealand rabbit Achilles tendon injury model and found that injection of bFGF at the damaged area improved biomechanical resistance and collagen fiber orientation. However, the number of fibroblasts and angiogenesis did not greatly change.

In conclusion, these studies demonstrate the positive effect of exogenous bFGF on tendon healing by promoting angiogenesis, cell proliferation, and collagen production, as well as by increasing the biomechanics of the repaired tendon.

## The Mechanisms of Basic Fibroblast Growth Factor in Tendon Healing

**FIGURE 2:**
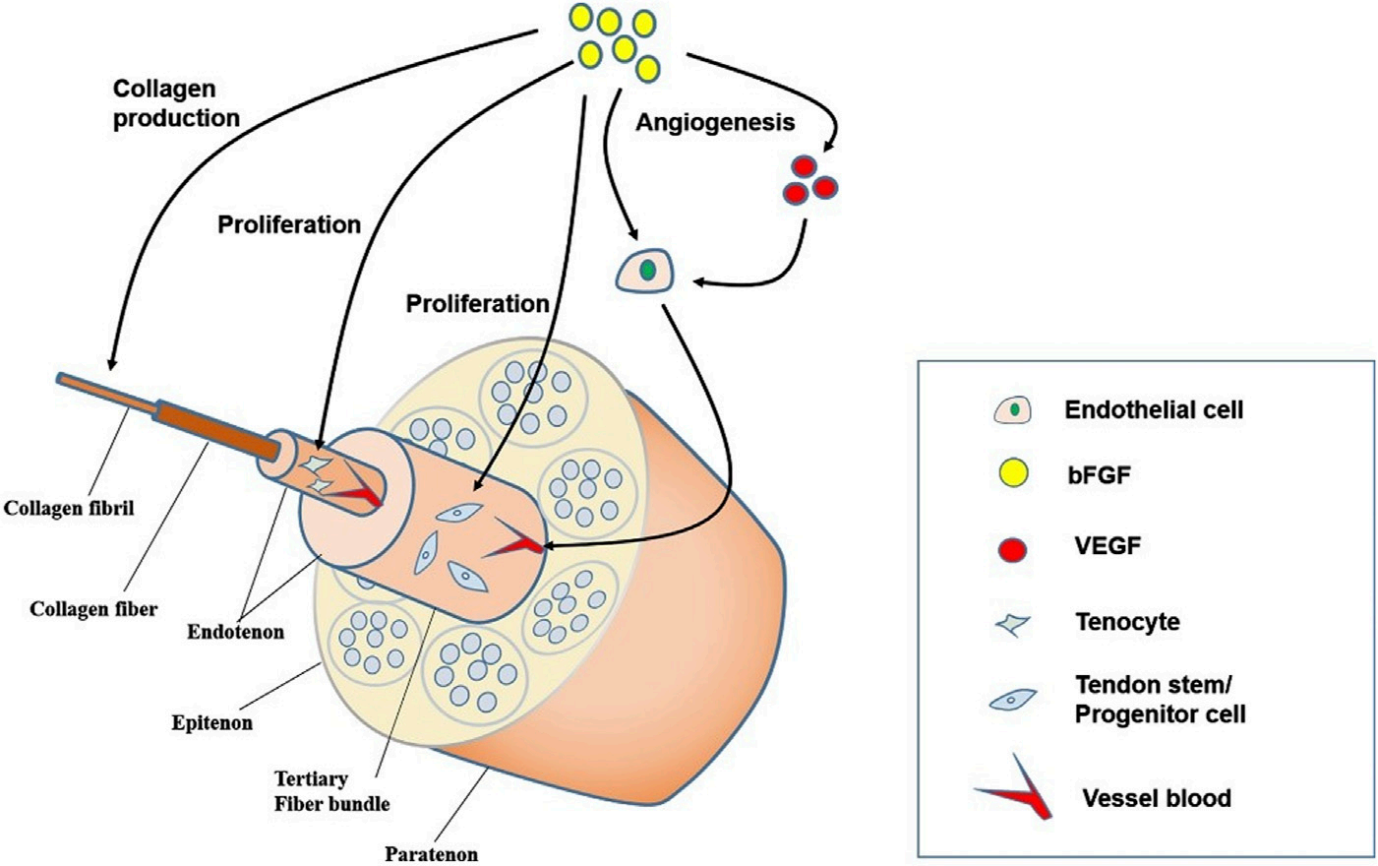
Mechanisms of the role of bFGF in the process of tendon healing. bFGF promotes angiogenesis, TSCs and tendon cell proliferation, as well as collagen production, thereby promoting tendon repair. bFGF, Basic fibroblast growth factor; VEGF, vascular endothelial growth factor; TSCs, tendon stem cells.

The tendon healing process is subdivided into three chronological stages, which are integrated with each other and not independently separated: the inflammatory phase, the cell proliferation phase, and the remodeling phase (Sharma and Maffulli, 2005). The initial inflammatory phase starts with a formation of a hematoma immediately after the injury (Hope and Saxby, 2007). When platelets chemotactically are activated to attract leucocytes, the latter can migrate into the injured tissue and produce bFGF, which accelerates the aggregation of inflammatory cells (Evans, 1999; Yang et al., 2013; Snedeker and Foolen, 2017a). Then monocytes differentiate into macrophages, with macrophages engulfing some of the necrotic cells and activating tendon cells, which act together at the site of injury (Yang et al., 2022). After a few days, the cell proliferation phase begins, with fibroblasts and endogenous tenocytes act together on the damage area, where there is more type III collagen than type I collagen (Leadbetter, 1992; Leong et al., 2020; Sang et al., 2020). bFGF can enhance type III collagen synthesis in the early phase of tendon healing, while it can promote type I collagen synthesis in the later phase. The remodeling period lasting longer, months or years, when the tendon cells and collagen fibers are aligned in the direction of stress in order to restore the toughness and tensile strength of the tendon (Leadbetter, 1992). At this stage, type III collagen gradually transforms to type I collagen and fibroblasts gradually differentiate into myofibroblasts (Wu et al., 2017; Best et al., 2019). bFGF plays a crucial function in all three phases of tendon healing, and its molecular effects on tendon structure are depicted in Figure 2.

### Basic Fibroblast Growth Factor Modulates the Inflammatory Process

The inflammatory response is a necessary component for tendon healing. Whilst acute and controlled inflammation is beneficial to the tissues, chronic and uncontrollable inflammation is thought to drive fibrosis (Tang et al., 2018). Tendon injuries are characterized by the production of massive amounts of inflammatory mediators, and if the tendon remains in the inflammatory phase for a long time, chronic tendinopathy can be developed, which has side effects on the tendon (Dakin et al., 2014). Previous studies have reported that bFGF increases significantly in the early stages of tendon repair, not only for their ability to rapidly accumulate inflammatory cells, but also for their chemotactic effect on pro-inflammatory factors (Zittermann and Issekutz, 2006).

After tendon injury, inflammatory cells such as neutrophils, macrophages and mast cells are recruited through the activation of some immune cell then migrating from the surrounding tissues to the site of injury (Butler et al., 2004). Fibroblasts and macrophages, as well as neutrophils, are capable of releasing bFGF, which absorb more pro-inflammatory factors, such as IL-1β and TNF-α to the site of injury, accelerating the inflammatory response. Macrophages have both pro-inflammatory and anti-inflammatory effects, and bFGF enhances the recruitment of macrophages (Chang et al., 1998). Macrophages, when activated, can differentiate into two types of cells, one is M1, which is the proinflammatory cell, and the other is M2, which is the proinflammatory repair cell. M1 cells have an important role in promoting the inflammatory process, and M2 cells have an essential effect in the later stages of inflammatory tendon healing (Sunwoo et al., 2020; Yang et al., 2022). Although bFGF is not directly involved in the anti-inflammatory response, it can indirectly participate in the anti-inflammatory response by enhancing cell adhesion molecules (CAMs) on endothelial cells (Zittermann and Issekutz, 2006). In addition, it has been shown that during the acute inflammatory phase, tendon damage is accompanied by pain, while bFGF can reduce pain by decreasing the concentration of profound prostaglandin E2 (PGE2), as well as reducing the concentration of nitric oxide and oxygen free radicals to maintain the viscoelasticity of tendons (Hu et al., 2009). The roles of inflammatory mediators and immune cells in tendon injury as shown in Table 2.

**TABLE 2:** The role of inflammatory mediators and immune cells in tendon injury IL-1β, Interleukin-1β; TNF-α, tumor necrosis factor-α; PGE2, prostaglandin E2.

Name	Functions
IL-1β	• IL-1β produce pro-inflammatory factors and promote the degradation of ECM
TNF-α	• Stimulates the production of pro- and anti-inflammatory factors by tenocytes, while matrix metalloproteinases can be produced
Macrophages	• M1 macrophages promote inflammatory responses
	• M2 macrophages dampen the inflammatory response and stimulate neovascularization
Mast cells	• Release of mediators to activate immune cells and promote neovascularization
PGE2	• Regulation of matrix metalloproteinase secretion, inhibiting inflammatory response, causes pain and promotion of angiogenesis

Inflammation, although an integral part of the tendon repair process, can boost the production of granulation tissue at the site of injury, which in turn can stimulate cell proliferation (Vinhas et al., 2018). However, high levels of pro-inflammatory cytokines and an uncontrolled inflammatory response are detrimental to tendon healing. Therefore, control of the inflammatory response is of great importance.

### Basic Fibroblast Growth Factor Speeds up Angiogenesis

Due to the fact that there are few blood vessels around normal tendon tissue, the healing rate is very slow when the tendon is injured (Mienaltowski and Birk, 2014). A multitude of evidences suggest that bFGF stimulates angiogenesis during tendon healing, and the new blood vessels have a role in delivering nutrients and oxygen as well as transporting metabolites (Springer et al., 2008; Yu et al., 2017; Elbialy et al., 2020).

bFGF is a mitogenic growth factor that induces not only the proliferation and differentiation of endothelial cells, but also the production of vascular endothelial growth factor (Tang et al., 2016). One of the mechanisms by which bFGF promotes angiogenesis is that it stimulates endothelial cells to secrete collagenases that lead to degradation of the vascular basement membrane, followed by the invasion of surrounding cells into the underlying cytoplasmic matrix and the formation of a capillary network, thereby promoting neovascularization and accelerating the healing of tendon injuries (Chang et al., 1998). Thomopoulos et al., implanted a fibrin matrix with a heparin delivery system containing bFGF into an injured flexor tendon in a dog, and after 21 days of repair, evaluated the histology of the tendon and observed increased neovascularization (Thomopoulos et al., 2010).

Moreover, another mechanism by which bFGF facilitates vascular growth is that it can upregulate the expression of vascular endothelial growth factor (VEGF), which then indirectly promotes angiogenesis (Tang et al., 2014). VEGF is a specific pro-angiogenic factor that increases the permeability of blood vessels (Bao et al., 2009). According to the literature, VEGF is not expressed in normal tendons and only after tendon injury, but endogenous VEGF expression in injured tendons is not sufficient to cause recovery from tendon injury (Liu et al., 2021). Meanwhile, during the angiogenesis phase, bFGF can work in combination with vascular endothelial growth factor to promote neovascularization together (Xing et al., 2020). Just by applying plasmid DNA containing the genes encoding VEGF164 and FGF2 to horses with tendinopathy, as in the study by Milomir Kovac et al., VEGF and bFGF were found to interact synergistically to stimulate angiogenesis (Kovac et al., 2018).

In conclusion, bFGF can speed up the formation of new blood vessels, thereby accelerating the healing rate of tendons. Although angiogenesis is extremely critical in the process of tendon healing, However, studies have shown that chronic tendon injuries are often accompanied by increased neovascularization, when VEGF-induced neovascularization hinders tendon healing (Fenwick et al., 2002; Pufe et al., 2005). Although both VEGF and bFGF are crucial pro-angiogenic factors, bFGF does not play a major role in pathological angiogenesis. For example, only high VEGF expression was detected in the blood serum of individuals with rotator cuff injury, and bFGF was not up-regulated significantly (Savitskaya et al., 2011). bFGF needs to be further investigated in future experiments to determine whether it impedes the healing of chronic tendon injuries.

### Basic Fibroblast Growth Factor Promotes Cell Proliferation

Tenocytes are the basic functional unit of the tendon which are slow to proliferate due to their being a highly differentiated cell. Meanwhile, one of the reasons for the difficulty of tendon healing is that tenocytes make up only 5% of the tendon tissue mass (Bi et al., 2007; Lui and Chan, 2011). In addition, studies in recent years have shown that another type of cell, the tendon stem cells (TSCs), is also present in the tendon tissue and that it has the capability to convert into tenocytes (Viganò et al., 2017). (Kraus et al., 2016a).

It has been suggested that one of the mechanisms by which bFGF promotes tenocytes proliferation and differentiation is that it can induce TSCs to differentiate into tendon cells (Kraus et al., 2016b). TSCs are cells with specific functions, which have a strong ability of self-renewal and differentiation. bFGF can inhibit the conversion of TSCs to non-tendon cells, thus enhancing tendon healing (Wang and Nirmala, 2016; Viganò et al., 2017). There is one more point, I should touch on, that bFGF enhances the proliferation and differentiation of tendon cells directly, which can accelerate the conversion from C1 to S1 phase of cell division by facilitating the synthesis of cellular DNA. In a rat patellar tendon assay, different concentrations of bFGF were injected into the injured patellar tendons of rats, with cell numbers and type III collagen content assessed on days 7 and 14, respectively (Chan et al., 2000). The outcome demonstrated that not only the number of cells was increased in the group adding bFGF, but also the number of cells and the dose of bFGF were positively correlated.

The last but not the least, studies have indicated that the nuclear factor kappa-B (NF-κB) gene is activated by bFGF, which results in cell proliferation and collagen synthesis (Tang et al., 2003; Best et al., 2020). The NF-κB signaling pathway is associated with both inflammation and cell proliferation, greatly influence the outcomes of tendon healing (Xuan et al., 2016). Tang, et al. investigated the effect of applying bFGF on tenocytes obtained from rabbit intrasynovial tendon segments transplantation cultures. The results of the study showed that bFGF significantly promoted the expression of type I collagen and NF-κB genes, and the gene expression was positively correlated with the concentration of bFGF. In conclusion, this research suggests that bFGF stimulates the proliferation of tenocytes by activating NF-κB gene genes (Tang et al., 2003).

### Basic Fibroblast Growth Factor Stimulates Collagen Synthesis

A multitude of experiments *in vitro* and animal have demonstrated that the vital role of bFGF on collagen synthesis. The extracellular matrix (ECM) of normal tendon tissue consists of collagen fibers, elastic fibers, matrix (glucosaminoglycans, glycosaminoglycans, etc.) and of several inorganic substances. As we all know, fibroblasts are essential cells for the regulation of ECM components since they secrete the components that make up the ECM (Xie et al., 2008). As a powerful mitogenic growth factor, bFGF mediates collagen synthesis by encouraging the proliferation of fibroblasts (Chan et al., 2000; James et al., 2008). Collagen fibers are the major element of the ECM, which accounts for about 75% of the overall tendon mass, and elastic fibers account for about 2% of the overall tendon mass (Kannus, 2000). The most abundant collagen connective tissue in normal tendons is type I collagen, whereby tendons containing type I collagen have a high tensile strength. Type III collagen is more abundant in the early stages of tendon repair, and as tissue healing progresses, type III collagen is gradually replaced by type I collagen, a process accelerated by bFGF (Chen et al., 2008).

Yan et al. prepared a double-layer composite membrane EMI-PLVB, which releases Ibuprofen from the outer layer and bFGF from the inner layer. An Achilles tendon adhesion model was established using the right hind limbs of rats, and the middle of the Achilles tendon was transected and EMI-PLVB was wrapped around the repair site, followed by suturing with modified Kessler suture. Compared with double membranes without bFGF, double membranes loaded with bFGF promote the matures and differentiation of type I and type III collagen, and the healing site has regular collagen arrangement (Yan et al., 2021). In another study, adeno-associated virus containing the bFGF gene was transfer to tendon cells, and both type I collagen gene and type III collagen gene were expressed at increased levels compared to tendon cells without gene transfer. This indicates that bFGF can contribute to the synthesis of collagen in tendon cells (Wang et al., 2005). However, excessive collagen synthesis is the primary cause of scar formation. Therefore, it is vital that type III collagen is promptly converted to type I collagen.

ECM remodeling is a complex process that involves the breakdown of existing ECM proteins along with the synthesis and deposition of new ECM proteins (Bosman and Stamenkovic, 2003; Jones et al., 2006). bFGF enhances the remodeling of the extracellular matrix, and multiple cell growth factors are involved in this process. Collagen synthesis is accompanied by degradation of collagen by matrix metalloproteinases (MMPs) to prevent excessive deposition of ECM and formation of scar tissue (Leong et al., 2020). Studies have revealed that bFGF can be used to enhance collagen degradation by promoting the expression of cellular MMPs, improving the alignment of collagen fibers, preventing the formation of scar tissue, in the end contributing to tendon healing.

## Conclusions and Perspectives

Tendon injuries result in a relatively high morbidity, and can be separated into acute and chronic injuries (Sharma and Maffulli, 2005). When an acute injury is not healed for a prolonged period of time, it will largely develop into a chronic injury (Aicale et al., 2018). In recent years, growth factors have been a popular treatment for tendon injuries, which provides new thoughts on the healing of tendon injuries (Kataoka et al., 2021). It was confirmed that the combination of multiple growth factors had superior efficacy compared with individual growth factors (Thomopoulos et al., 2005; Kobayashi et al., 2006). For instance, the combination of bFGF, bone morphogenetic protein (BMP-12), and transforming growth factor beta 1 (TGFβ_1_) was used in a study to remedy tendon injuries. Majewski1 et al. implanted collagen sponges containing the growth factors bFGF, BMP-12, and TGFβ_1_ into transected rat Achilles tendons. The findings showed that the healed tendon load and tendon stiffness were excellent compared to the control group, in addition with thicker collagen fiber bundles and more vascular distribution (Majewski et al., 2018). Firstly, bFGF has advantages in angiogenesis and collagen synthesis. Secondly, BMP-12 can increase the production of type I collagen, while TGFβ not only stimulates tendon cell migration and production of ECM, but also transforms type III collagen into type I collagen (Lou et al., 2001; Subramanian et al., 2018). The combination of all three growth factors can enhance tendon healing at different stages and reduce the time to healing and improve the quality of tendon healing (Majewski et al., 2018). In future research areas more attention should be paid to the potential of combined growth factors for therapy, where multiple growth factors can modulate tendon healing through diverse time points and various processes.

A variety of studies have shown that bFGF has a positive effect on the tendon repair process and that the addition of exogenous bFGF not only synergizes endogenous bFGF, but also spurs the production of other cellular growth factors. bFGF contributes to tendon healing by enhancing neovascularization, cell proliferation and collagen synthesis, and it also regulates the growth of fibroblasts. During the inflammatory phase, bFGF both reduces pain at the damaged area and promotes the inflammatory response. When injecting higher concentrations of exogenous bFGF into the damaged area, the number of cells does not improve with increasing concentration, demonstrating that bFGF fosters cell proliferation directly, rather than through chemotaxis.

As a matter of fact, except for promoting tendon healing, bFGF can also relieve post-healing adhesions as well as enhance the biomechanical strength of the healed tendon. Shen et al. found that transected New Zealand rabbit Achilles tendons treated with focused ultrasound with targeted bFGF plasmid-loaded cationic microbubbles showed superior wound healing, smooth Achilles tendons, and non-adhesion to surrounding tissues (Shen et al., 2020). Similarly, Yonemitsu et al. established a model of rotator cuff (RC) tear in rats, which was surgically fixed after 3 weeks, followed by implantation of gelatin hydrogel containing FGF-2 between the tendon end and the bone. They demonstrated that the biomechanical strength of the injured supraspinatus tendon in rats treated with FGF-2 for 6 and 12 weeks was significantly improved. In addition to this, the expression of tendon-related genes including scleraxis (SCX) and tenomodulin (Tnmd) was increased in the FGF-2 treated group, which contributed to the formation of tendon-like tissue (Yonemitsu et al., 2019). One of the mechanisms by which FGF-2 enhances the biomechanical strength of tendon bone healing is through increased production of mesenchymal progenitor cells, and the other is by promoting the expression of SCX and Tnmd (Tokunaga et al., 2015b). Likewise, (Zhou et al., 2021) established two models, a chicken model of flexor tendon injury and a rat model of Achilles tendon injury, both with bFGF/VEGFA loaded nanoparticle-coated sutures for circular repair at the stump of the injured tendon. The results showed that bFGF/VEGFA loaded nanoparticle-coated sutures not only promoted tendon healing and the ultimate strength of the repaired tendon, but also prevented tendon adhesions.

**FIGURE 3:**
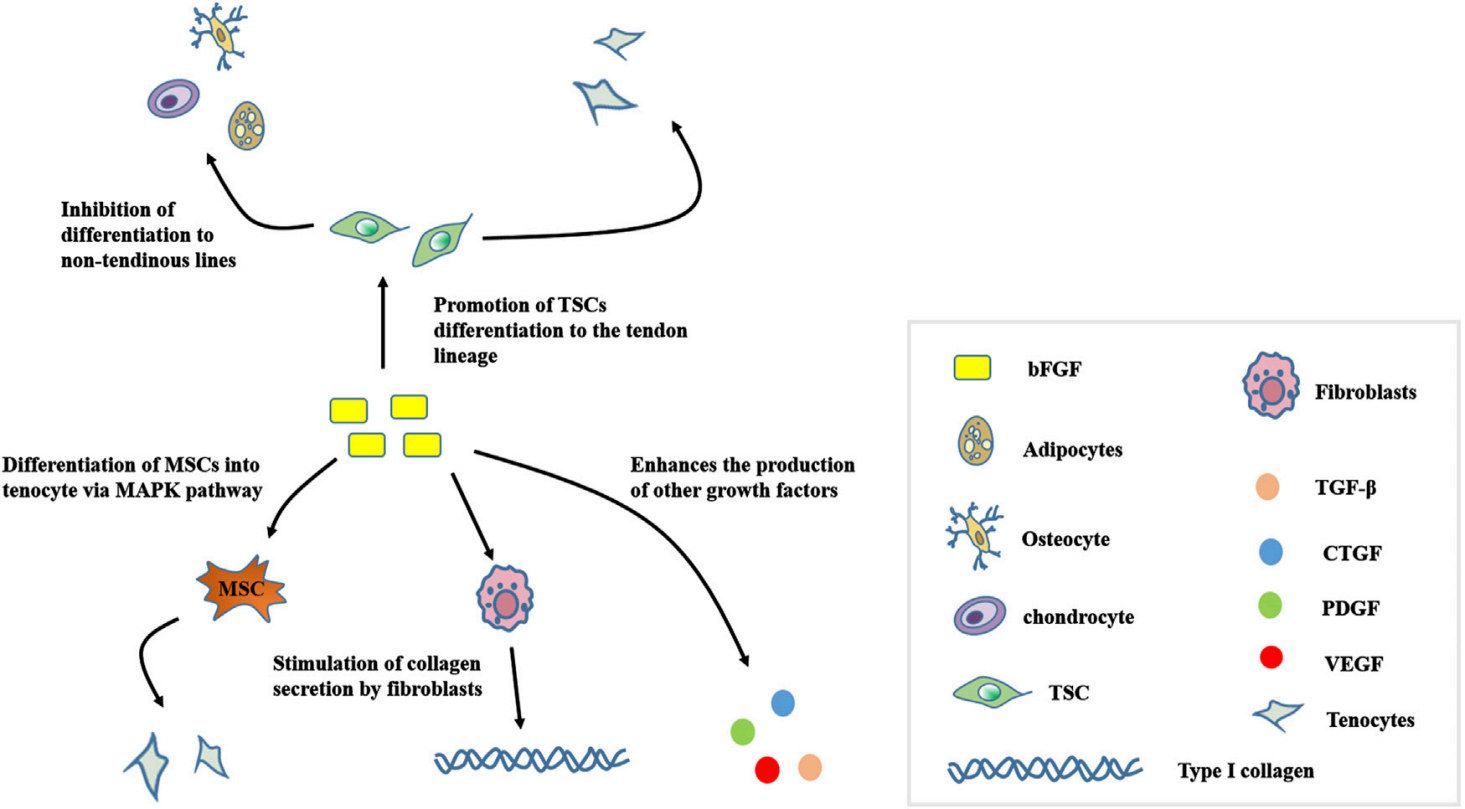
Mechanism of action for bFGF in tendon stem cells, mesenchymal stem cells, and fibroblasts. bFGF, Basic fibroblast growth factor; TSC, tendon stem/progenitor cell; TGF-β, transforming growth factor beta; CTGF, Connective tissue growth factor; PDGF, platelet-derived growth factor; VEGF, vascular endothelial growth factor.

*In vitro* animal studies have shown that bFGF is a powerful mitotic cell growth factor that multiplies and differentiates mesenchymal stem cells (MSCs) into tendon cells *via* the MAPK pathway (Cai et al., 2013). TSCs have been recently identified in mice and humans as well as have multifunctional differentiation potential, but inappropriate stimulation can cause conversion of TSCs to non-tendon lineages and affect the tendon healing process (Zhang and Wang, 2010). Experiments have shown that the addition of exogenous bFGF to TSCs led to their proliferation and differentiation into tendon cells (Viganò et al., 2017). Figure 3 shows the role of bFGF on TSCs, MSCs, as well as fibroblasts and other growth factors. bFGF treatment of tendon injuries also has the disadvantage that excessive use of bFGF can result in adhesions to the tendon, thus affecting the function of the tendon after recovery. It is extremely rare for the healed tendon to return to the structural and mechanical properties of a healthy tendon and are prone to re-injury. Although there are studies showing that bFGF improves the biomechanical function of healed tendons, opposite researches have disproved this conclusion (Thomopoulos et al., 2010; Moshiri and Oryan, 2011).

Since growth factors have a short half-life in the body, repeated injections of growth factors are required, which is more time-consuming and laborious, so some delivery vehicle for growth factors is needed to enable their sustained release, such as scaffolds in tissue engineering (Andreopoulos and Persaud, 2006; Zhao et al., 2014). To illustrate, Liu et al. electrospun dextran glassy nanoparticles (DGNs) containing bFGF into poly-L-lactic acid (PLLA) copolymer fibers, called bFGF/DGNs-PLLA membrane, which has the advantage of sustained release of bFGF. They established a rat model of Achilles tendon transection with bFGF/DGNs- PLLA membrane to wrap the tendon repair site. The results showed that the bFGF/DGNs-PLLA membrane promoted tendon healing while preventing adhesions (Liu et al., 2013). Additionally, the core of tendon tissue engineering is the construction of tendon cell lines. Stem cells are a promising cell for seeding, and bFGF promotes the proliferation and differentiation of MSCs on poly (lactide-co-glycolide) (PLGA) scaffolds, as well as the generation of neovascularization (Zhao et al., 2019). Tissue engineering holds significant promise for tendon regeneration; however, future researches are needed to optimize the application of bFGF in tissue engineering.
